# Tick Humoral Responses: Marching to the Beat of a Different Drummer

**DOI:** 10.3389/fmicb.2017.00223

**Published:** 2017-02-14

**Authors:** Adela S. Oliva Chávez, Dana K. Shaw, Ulrike G. Munderloh, Joao H. F. Pedra

**Affiliations:** ^1^Department of Microbiology and Immunology, University of Maryland School of Medicine, BaltimoreMD, USA; ^2^Department of Entomology, University of Minnesota, Saint PaulMN, USA

**Keywords:** tick-borne diseases, Lyme disease, vector, ticks, humoral immunity

## Abstract

Ticks transmit a variety of human pathogens, including *Borrelia burgdorferi*, the etiological agent of Lyme disease. Multiple pathogens that are transmitted simultaneously, termed “coinfections,” are of increasing importance and can affect disease outcome in a host. Arthropod immunity is central to pathogen acquisition and transmission by the tick. Pattern recognition receptors recognize pathogen-associated molecular patterns and induce humoral responses through the Toll and Immune Deficiency (IMD) pathways. Comparative analyses between insects and ticks reveal that while the Toll pathway is conserved, the IMD network exhibits a high degree of variability. This indicates that major differences in humoral immunity exist between insects and ticks. While many variables can affect immunity, one of the major forces that shape immune outcomes is the microbiota. In light of this, we discuss how the presence of commensal bacteria, symbionts and/or coinfections can lead to altered immune responses in the tick that impact pathogen persistence and subsequent transmission. By investigating non-insect arthropod immunity, we will not only better comprehend tick biology, but also unravel the intricate effects that pathogen coinfections have on vector competence and tick-borne disease transmission.

## Introduction

Ticks are increasingly important disease vectors that transmit a variety of pathogens relevant to public and veterinary health (de la Fuente et al., 2008; Stromdahl and Hickling, 2012; Hartemink and Takken, 2016; Kernif et al., 2016). The most prevalent vector-borne illness in the Northern hemisphere, Lyme disease, is transmitted by *Ixodes* spp. ticks and is caused by the spirochete *Borrelia* spp. (Mather and Mather, 1990). Ticks are first colonized by pathogens when they take a bloodmeal from an infected host. The microbes will then lie dormant throughout digestion and molting. Subsequent transmission to a new vertebrate host occurs during the second bloodmeal, where pathogens migrate to the salivary glands and are injected along with saliva. Multiple obstacles within the vector can impact pathogen survival and persistence (Liu and Bonnet, 2014), including the arthropod’s immune system. This is the foremost defense against invading microbes and largely impacts the ability of an arthropod to be a competent vector for pathogens (Hillyer et al., 2003; Garver et al., 2009; Blumberg et al., 2013).

Arthropod immunity lacks adaptive components and is limited to innate processes, which can be categorized as either cellular or humoral (Ganesan et al., 2011; Buchon et al., 2014; Myllymaki et al., 2014). Humoral immunity involves innate signaling cascades, such as the Toll and Immune Deficiency (IMD) pathways. Immune defenses are triggered by pathogen-associated molecular patterns (PAMPs), which are sensed by pattern recognition receptors (PRRs) (Hillyer, 2016). Both pathogenic and commensal bacteria can elicit immune responses in arthropods, which makes the composition of the microbiota a significant force in determining vector competence as well (Cirimotich et al., 2011). For the purposes of this article, the microbiome/microbiota will be defined as all microorganisms present in the arthropod including symbionts, commensals and pathogens.

Although insect immunity has been heavily studied and is well understood, owing to the model organism *Drosophila melanogaster*, recent data demonstrates that non-insect arthropods, such as ticks, are significantly different (Palmer and Jiggins, 2015; Gulia-Nuss et al., 2016; Rosa et al., 2016; Shaw et al., 2017). Genome sequencing data shows that ticks lack several genes involved in innate immunity when compared to insects including some PRRs, pathway signaling molecules and antimicrobial peptides (AMPs) (Severo et al., 2013; Smith and Pal, 2014; Palmer and Jiggins, 2015; Bechsgaard et al., 2016; Gulia-Nuss et al., 2016; Rosa et al., 2016). Nevertheless, immune pathways within ticks remain functional, suggesting that there are undiscovered principles governing non-insect arthropod immunity (Kopacek et al., 1999; Sonenshine et al., 2002; Simser et al., 2004; Pelc et al., 2014). Herein, we will discuss the current understanding of tick humoral signaling pathways in the context of disease transmission both with and without confounding factors, such as coinfections and the microbiota.

## Humoral Immunity

Two of the best studied immune signaling cascades in arthropod immunity are the Toll and IMD pathways. Both are initiated by distinct PAMPs and orchestrate the production of microbiocidal AMPs (Hillyer, 2016). The Toll pathway responds primarily to Gram-positive bacteria and fungi whereas the IMD pathway recognizes Gram-negative bacteria (Hillyer, 2016). Herein, we will describe our current understanding of tick humoral immunity in comparison to insects.

### The Toll Pathway

**FIGURE 1:**
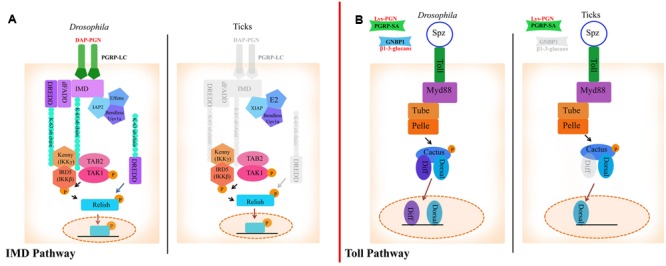
**The Immune Deficiency (IMD) and Toll pathways in *Drosophila* and ticks. (A)** Activation of the IMD pathway in *Drosophila* is initiated by PGRP-LC binding to diaminopimelic acid (DAP)-type peptidoglycan. This leads to IMD, dFADD, and DREDD recruitment. IMD is cleaved by DREDD, exposing an ubiquitylation site and is polyubiquitylated by IAP2, Effete, Uev1a and Bendless in a K63-dependent manner. K-63 polyubiquitin chains are believed to serve as the recruiters for the proteins TAB2, TAK1, and IKK (IKKγ and IKKβ), which transfer a phosphate group to Relish. Relish is then cleaved by DREDD, removing the C-terminal ankyrin repeats. The N-terminal portion of Relish is translocated to the nucleus where it induces the transcription of AMPs (Vandenabeele and Bertrand, 2012). In ticks, transmembrane PGRPs, IMD, dFADD and possibly DREDD are missing (shaded gray). XIAP is suggested to regulate the IMD pathway in ticks through direct interaction with Bendless (Shaw et al., 2017). **(B)** In *Drosophila*, the Toll pathway is activated by PGRPs and GNBPs binding to Lysine-type peptidoglycan or β1-3-glucan, respectively. PAMP binding to PRRs leads to activation of ModSP (Modular Serine Protease) and Grass in the extracellular milieu. Spz is then cleaved and binds to Toll receptors. Following Spz binding, MyD88 dimers interface with the Toll receptor and recruit Tube, an adaptor molecule that interacts with the protein kinase Pelle. Cactus is then phosphorylated and degraded, which leads to translocation of Dif (Dorsal-related immunity factor) and/or Dorsal to the nucleus and AMP upregulation (Lindsay and Wasserman, 2014). The tick genome encodes all components of the Toll pathway, with the exception of *GNBPs* and *dif*.

In *Drosophila*, Lysine-type peptidoglycan from the cell wall of Gram-positive bacteria is recognized by peptidoglycan recognition receptor proteins (PGRPs)-SA. β1-3-glucan from fungi is sensed by Gram-negative binding proteins (GNBPs) (Michel et al., 2001; Kanagawa et al., 2011) (**Figure 1**). Most of the components that comprise the Toll pathway in insects are conserved in the tick genome, although there are a few deviations (**Figure 1**; **Table 1**) (Palmer and Jiggins, 2015; Bechsgaard et al., 2016). For example, there are eight Toll receptors found in *Drosophila*, whereas only four have been identified in the *Ixodes scapularis* genome (Palmer and Jiggins, 2015). *I. scapularis* ticks also lack genes encoding *GNBPs* (Palmer and Jiggins, 2015; Gulia-Nuss et al., 2016). Despite the reduction in receptor repertoire, evidence for functional Toll signaling in ticks exists. *In vitro* challenge of *Rhipicephalus microplus* with *Enterobacter cloacae, Micrococcus luteus* and *Saccharomyces cerevisiae* lead to upregulation of *toll, myD88, tube, pelle*, and *cactus* suggesting pathway functionality (Rosa et al., 2016).

**Table 1:** Arthropod humoral networks.

Pathway	Component	Insects			Crustacea		Arachnids		
		*Drosophila*^α^	*Anopheles*^β,𝜀^	Pea aphid^𝜃^	*Daphnia* spp.^α,𝜀^	Shrimp^ζ^	Mites^α^	*Ixodes* spp.^α,δ^	*Rhipicephalus* spp.^δ^
Toll Pathway	PGRP	+	+	-	-	-	+	+	+
	GNBP	+	+	+	+	-	-	-	-
	Spz	+	+	+	+	+	+	+	+
	Toll	+	+	+	+	+	+	+	+
	MyD88	+	+	+	+	+	+	+	+
	Tube	+	+	+	-	-	-	+	+
	Pelle	+	+	+	+	+	+	+	+
	Dif/Dorsal	+	+	+	+	+	+	+	+
	Cactus	+	+	+	+	+	+	+	+
IMD pathway	Transmembrane PGRPs	+	+	-	-	-	-	-	-
	Soluble PGRPs	+	+	-	-	-	+	+	+
	IMD	+	+	-	+	+	-	-	-
	dFADD	+	+	-	+	-	-	-	-
	DREDD	+	+	-	+	-	-	-	-
	IAP2	+	+	+	+	+	+	+	+
	Bendless	+	+	+	+	+	+	+	+
	Uev1a	+	+	+	+	+	+	+	+
	Effette	+	+	+	+	+	+	+	+
	XIAP	+	+	+	+	+	+	+	+
	TAB2	+	-	+	-	-	-	+	+
	TAK1	+	+	+	+	-	+	+	+
	IKKγ	+	+	-	+	-	-	+	+
	IKKα/β	+	+	+	+	+	+	+	+
	Relish	+	+	-	+	+	+	+	+
	Caspar	+	+	+	+	+	+	+	+
	Caudal	+	+	+	+	+	+	+	+

*α = (Palmer and Jiggins, 2015); β = (Waterhouse et al., 2007); 𝜃 = (Gerardo et al., 2010); δ = (Rosa et al., 2016); 𝜀 = (McTaggart et al., 2009); ζ = (Liu et al., 2009; Li and Xiang, 2013; Udompetcharaporn et al., 2014).*

*Drosophila* transcriptional regulators controlled by the Toll pathway, Dif and Dorsal, regulate the expression of *defensin* and other AMPs (Meng et al., 1999). Interestingly, instances of cooperation between transcription factors have been described (Meng et al., 1999). Optimal induction of *defensin* was reported when the IMD pathway-regulated transcription factor, Relish, formed heterodimers with Dif or Dorsal (Han and Ip, 1999). These experiments were performed *in vitro* with stably transfected cell lines and thus the *in vivo* relevance is unclear, but suggests interesting potential for defenses orchestrated by multiple immune pathways. Ticks also produce several Defensin-like AMPs (Johns et al., 2001b; Sonenshine et al., 2002; Ceraul et al., 2003, 2007; Lai et al., 2004; Hynes et al., 2005; Zhou et al., 2007; Wang and Zhu, 2011; Chrudimska et al., 2014; Pelc et al., 2014). Although the mechanism of *defensin* regulation in ticks is not characterized, the highly conserved nature of the Toll pathway suggests that it may act similarly to insects. Moreover, tick Defensins are secreted in response to both Gram-positive and negative bacteria, suggesting that there may be a similar mechanism of cross-talk in non-insect arthropods (Sonenshine et al., 2002).

### The IMD Pathway

Diaminopimelic acid (DAP)-type peptidoglycan from Gram-negative bacteria stimulates the IMD pathway in *Drosophila*, which is recognized by both transmembrane and soluble PGRPs (Boutros et al., 2002; Hillyer, 2016). Ticks lack several key components of the IMD pathway such as transmembrane PGRPs, *imd, dFADD*, and IMD pathway-specific AMPs (**Table 1**; **Figure 1**) (Severo et al., 2013; Palmer and Jiggins, 2015; Gulia-Nuss et al., 2016; Rosa et al., 2016). Despite lacking key components, the IMD pathway is functional in ticks (Shaw et al., 2017). The *I. scapularis* Relish is activated in response to *Anaplasma phagocytophilum* infection and knocking down regulatory components from the IMD pathway (*relish, capsar, uev1a*, and *bendless*) lead to altered pathogen burden levels with both *A. phagocytophilum* and *Borrelia burgdorferi* (Shaw et al., 2017). A separate study also showed that bacterial infection of *R. microplus* lead to transcriptional upregulation of IMD signaling components (*tak1, tab2, ikk*β, *ikk*γ, and *relish*) (Rosa et al., 2016). Taken together, these studies provide evidence for a functional IMD pathway in ticks.

*Drosophila* PGRP-LC and PGRP-LE are IMD pathway receptors (Kaneko et al., 2006) and PGRP-SD is an IMD co-receptor (Iatsenko et al., 2016). Transmembrane PGRP-LC and soluble PGRP-LE multimerize after binding to DAP-type peptidoglycan and initiate signaling by recruiting IMD to the RIP Homotypic Interaction Motif (RHIM) (**Figure 1**) (Kaneko et al., 2006). PGRP-SD, initially thought to activate Toll signaling (Bischoff et al., 2004), elicits the IMD pathway by interacting with PGRP-LC (Iatsenko et al., 2016) and DAP-type peptidoglycan (Leone et al., 2008). Although there are four encoded *PGRPs* in the *I. scapularis* genome, none are predicted to be transmembrane proteins or to have the IMD-interacting RHIM domain (Palmer and Jiggins, 2015). This is consistent with the lack of *imd* in the genome, suggesting an alternative mode of pathway activation (**Figure 1**) (Palmer and Jiggins, 2015; Bechsgaard et al., 2016). The role of secreted PGRPs in ticks is unknown, although a recent study showed that silencing the soluble *I. scapularis PGRPs* did not have a significant effect on *A. phagocytophilum* colonization (Shaw et al., 2017).

K63-dependent polyubiquitylation of IMD and dDREDD (Death related ced-3/Nedd2-like caspase) by the E3 ubiquitin ligase, inhibitor of apoptosis protein 2 (IAP2), is necessary for signal transduction in *Drosophila* (Paquette et al., 2010; Meinander et al., 2012). A different E3 ubiquitin ligase in ticks, X-linked inhibitor of apoptosis (XIAP), has been shown to influence *A. phagocytophilum* burden (Severo et al., 2013) by interfacing with the IMD pathway (Severo et al., 2013; Shaw et al., 2017). XIAP physically interacts with the IMD pathway E2 ubiquitin conjugating enzyme, Bendless, and carries out K63-dependent polyubiquitylation together with Uev1a (Shaw et al., 2017). Moreover, double knockdown of *bendless-uev1a* heterodimers and *xiap* lead to increased colonization by both *A. phagocytophilum* and *B. burgdorferi*, suggesting a defect in pathogen control (Shaw et al., 2017).

In addition to alternative signaling modes, there is evidence that PAMPs other than DAP-type peptidoglycan can trigger the IMD signaling cascade. Reports of virus and parasite-induced IMD pathway activation in insects lend support to this hypothesis (Baton et al., 2009; Costa et al., 2009). In ticks, the IMD circuitry senses infection-derived lipids 1-palmitoyl-2-oleoyl-sn-glycero-3-34 phosphoglycerol (POPG) and 1-palmitoyl-2-oleoyl diacylglycerol (PODAG), and leads to Relish activation (Shaw et al., 2017). Moreover, priming ticks with these lipids induced protection against *A. phagocytophilum* and *A. marginale* infection both *in vitro* and *in vivo*, respectively (Shaw et al., 2017). These findings coupled with the lack of transmembrane PGRPs and key signaling molecules suggest that a non-canonical IMD pathway exists in ticks.

## Other Immune Signaling Pathways

The Janus Kinase/Signal Transducer and Activator of Transcription (JAK/STAT) pathway is not part of the humoral innate response in insects, but does have a role in immunity through crosstalk with IMD and Toll signaling (Myllymaki and Ramet, 2014). The JAK/STAT pathway is activated by the receptor Dome through recognition of the cytokine signaling molecule, Unpaired (Upd) (Harrison et al., 1998; Brown et al., 2001). This interaction results in phosphorylation of Hop proteins and translocation of Stat92E to the nucleus, which stimulates expression of cytokines and members of the *tot* family (Harrison et al., 1995; Agaisse et al., 2003; Bach et al., 2003; Lamiable et al., 2016). The *I. scapularis* JAK/STAT pathway is important for the control of *A. phagocytophilum* and regulates the expression of a gene family that encodes 5.3 kDa antimicrobial peptides (Liu et al., 2012). Comparative analysis demonstrates that the JAK/STAT pathway is well conserved between ticks and *Drosophila* (Palmer and Jiggins, 2015; Bechsgaard et al., 2016), with the exception of *upd* (Liu et al., 2012; Rosa et al., 2016).

Beyond pathogen control, the JAK/STAT pathway has an important role in physiological maintenance. *Drosophila* midgut homeostasis is influenced by the microbiota, which is regulated by phagocytic cells and the IMD pathway. This in turn impacts JAK/STAT signaling (Guillou et al., 2016). Mutation of either the CD36 phagocytic receptor, Croquemort, or Relish causes overexpression of Upd3 and dysregulated gut integrity, leading to increased mortality (Guillou et al., 2016). The mechanistic details involved in JAK/STAT activation in ticks are currently unknown, although the absence of *upd* is intriguing. A recent study showed that mammalian-derived interferon (IFN)-γ, present in the bloodmeal, stimulated the tick JAK/STAT pathway (Smith et al., 2016). This cytokine cross-talk upregulated the tick Rho-like GTPase (IGTPase) and induced expression of domesticated amidase effector (DAE2), an AMP homologous to eukaryotic effectors that hydrolyzes bacterial peptidoglycans (Chou et al., 2015; Smith et al., 2016). This is an interesting example of cross-species cytokine signaling and could indicate that midgut homeostasis in ticks and the microbiota are influenced by mammalian-derived signaling molecules.

## The Microbiome

### Non-symbiotic and Symbiotic Commensals

The microbiome is comprised of commensal bacteria in the gut and other endosymbionts (Narasimhan and Fikrig, 2015). Ticks harbor less complex microbial communities, likely due to their blood-only diet, than other vectors that are not exclusively hematophagous, such as mosquitoes (Hawlena et al., 2013; Clayton et al., 2015; Rynkiewicz et al., 2015). *Proteobacteria, Actinobacteria, Enterobacter, Sphingobacterium, Firmicutes, Pseudomonas*, and *Bacteroidetes* have all been associated with ticks, although bacterial composition varies depending on geographic region and sex (Van Treuren et al., 2015). Interestingly, there is evidence that the microbiota impacts the arthropod through involvement with the immune system (Hawlena et al., 2013). Commensal bacteria stimulate gut epithelium renewal through JAK/STAT signaling in *Drosophila* (Buchon et al., 2009). Similarly, the tick microbiota also impacts midgut epithelium and peritrophic membrane integrity (Narasimhan et al., 2014; Narasimhan and Fikrig, 2015).

Although blood is a nutrient-rich source, it lacks some metabolites that are essential for survival. Endosymbiotic relationships can provide these nutrients and have been observed in many hematophagous arthropods including tsetse flies, bed bugs, lice, reduviid bugs and ticks (Rio et al., 2016). For example, a *Coxiella-*like endosymbiont provides vitamins and co-factors to *Amblyomma americanum* ticks and is required for adequate fecundity (Smith et al., 2015). A combination of mechanisms is likely used to ensure balance between the arthropod and endosymbiont. The arthropod host must control endosymbiont numbers to avoid over stimulation of immune responses and/or nutrient deprivation. In contrast, endosymbiotic bacteria must evade or suppress immune recognition to avoid clearance (Herren and Lemaitre, 2011; Masson et al., 2015; Shokal et al., 2016). Limited information is known about these relationships, owing to the difficult nature of *in vitro* symbiont cultivation, although a few studies have been reported (Kurtti et al., 2015, 2016). For example, the intracellular *Dermacentor andersoni* endosymbiont, *R. peacockii*, is 150-fold more resistant to AMPs than extracellular bacteria, illustrating a mechanism of immune tolerance (Baldridge et al., 2005). Avoidance mechanisms remain largely understudied, but likely vary depending on the endosymbiont and tick host species.

### Pathogen Coinfection

Simultaneous colonization by multiple pathogens is termed “coinfection” and is becoming a major health concern worldwide (Steiner et al., 2008; Schulze et al., 2013). In Europe, over half of all surveyed *I. ricinus* ticks are coinfected (Moutailler et al., 2016), with the most prevalent instances occurring in areas that are forested and endemic for Lyme disease (Swanson et al., 2006). Coinfections can increase the severity of illness, as demonstrated with babesiosis and Lyme disease (Diuk-Wasser et al., 2016). Moreover, simultaneous infection of *Peromyscus leucopus* mice with the parasite, *Babesia microti*, and *B. burgdorferi* increased the number of parasites acquired by ticks during a bloodmeal. This was likely due to heightened parasitemia in the mouse during coinfection (Diuk-Wasser et al., 2016). Conflicting reports have been published about coinfections with *B. burgdorferi* and *A. phagocytophilum*. One study reported no observable differences in acquisition and transmission with *I. scapularis* ticks (Levin and Fish, 2000), whereas another demonstrated that *B. burgdorferi* burden in ticks increased when fed on mice coinfected with *A. phagocytophilum* (Thomas et al., 2001). *E. ruminantium* levels increased during *in vitro* coinfection with *B. burgdorferi* as well (Moniuszko et al., 2014). Importantly, coinfections are not a phenomenon limited to *Ixodes* ticks, as both *Rhipicephalus* sp. and *Hyalomma rufipes* ticks can harbor between two to four pathogens (Berggoetz et al., 2014). Taken together, this information suggests that coinfection is a previously unappreciated phenomenon that likely impacts tick-borne disease transmission and outcome.

## *B. burgdorferi* and Tick Immunity

**FIGURE 2:**
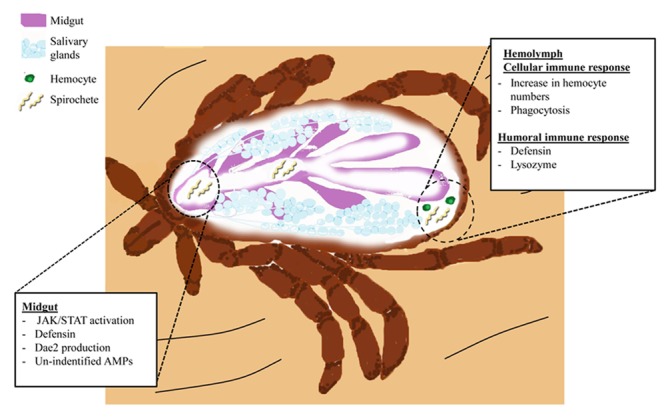
**The *I. scapularis* response to *B. burgdorferi* infection.** Spirochetes (light yellow) enter the tick midgut (purple) during blood feeding. Spirochetes interact with gut tissues and trigger activation of the JAK/STAT pathway. Induction of JAK/STAT signaling and possibly other pathways leads to AMP production (Defensins and DAE2). Spirochete migration into the hemolymph elicits cellular and humoral immunity. Cellular responses include increased prevalence of hemocytes (green) and initiation of phagocytosis. Humoral immunity results in the secretion of Defensins (originating from hemocytes and the fat body) and Lysozyme (hemocytes) into the hemolymph (Johns et al., 2001b; Ceraul et al., 2003, 2007). Niche-specific immune responses, such as those originating from the salivary glands (light blue structures), remain elusive.

Lyme disease is the most important vector-borne disease in the Northern hemisphere and approximately 30,000 cases are reported annually in the United States (Kugeler et al., 2015; Diuk-Wasser et al., 2016). *B. burgdorferi* colonizes ticks during a bloodmeal, where they will persist during digestion and molting (Radolf et al., 2012). Transmission subsequently occurs during a second bloodmeal when spirochetes are introduced into a new host with the saliva injected by a feeding tick (Radolf et al., 2012) (**Figure 2**).

Different species of ticks vary in their ability to transmit *Borrelia* spp. (Mather and Mather, 1990; Dolan et al., 1998). *Dermacentor* ticks, for instance, are not able to acquire or transmit *B. burgdorferi* (Mather and Mather, 1990). Spirochetes injected into *D. variabilis* are cleared from the hemocoel, whereas artificially infected *I. scapularis* retain the pathogen (Johns et al., 2001a). Inoculation of *B. burgdorferi* results in a rapid increase of hemocytes, lysozyme, and AMPs in *D. variabilis* (Johns et al., 2000; Sonenshine et al., 2002), which are likely major factors influencing this species’ competence. How and why these responses are not also induced in *Ixodes* ticks remains unknown, but is an intriguing topic.

The microbiome also influences vector competence. Ticks with a modified microbiota, termed “dysbiosed”, maintain lower *B. burgdorferi* numbers as compared to normal ticks (Narasimhan et al., 2014). Interestingly, this reduction in spirochetes appears to be related to midgut homeostasis and epithelial renewal controlled by JAK/STAT pathway-regulated expression of *peritrophin-1* (Narasimhan et al., 2014). A graphic representation of the humoral and cellular responses of ticks during *B. burgdorferi* infection can be found in **Figure 2**.

## Conclusion

Although ticks are of increasingly importance, little is known about what dictates their competence as disease vectors. It is known that immune networks heavily influence insect vector competence. However, there are fundamental differences in tick immunity when compared to insects. For example, the repertoire of Toll receptors found in ticks is reduced when compared to *Drosophila* (Palmer and Jiggins, 2015) and the IMD pathway has a significant amount of gene loss, yet both remain active (Severo et al., 2013; Smith and Pal, 2014; Palmer and Jiggins, 2015; Bechsgaard et al., 2016; Gulia-Nuss et al., 2016; Rosa et al., 2016). Unknown immune networks are likely present in ticks that facilitate the recognition of invading pathogens. Exploiting the long co-evolutionary history between ticks and the pathogens they can transmit, such as *Borrelia, Anaplasma, Ehrlichia*, and/or *Rickettsia*, is one avenue for approaching this gap in knowledge. For example, a non-canonical IMD network in ticks has recently been identified using both *A. phagocytophilum* and *B. burgdorferi* (Shaw et al., 2017).

Other confounding factors influencing pathogen transmission are coinfections and/or interactions with the microbiota. For instance, simultaneous infection of ticks with *A. phagocytophilum* and *B. burgdorferi* leads to higher spirochete burdens (Thomas et al., 2001). It is tempting to speculate that *A. phagocytophilum* virulence proteins exert an immunosuppressive effect on the tick that inadvertently confers a survival advantage for *B. burgdorferi*. Another point of interest is the recent evidence that mammalian-derived cytokines can cross-react with the tick immune system (Smith et al., 2016). This discovery sheds new light on what we know about vector competence because coinfection in the mammal will inevitably skew the cytokine profile of the host and thus the bloodmeal taken by a tick. Investigating alternative immune circuitry and agonists will not only lead to better understanding of tick biology and pathogen transmission, but will also illuminate how coinfections are maintained.

## Author Contributions

AO wrote this review. JP, UM, and DS contributed to intellectual discussions and editing of the article.

## Conflict of Interest Statement

The authors declare that the research was conducted in the absence of any commercial or financial relationships that could be construed as a potential conflict of interest.

The handling Editor declared a past co-authorship with one of the authors JP and states that the process nevertheless met the standards of a fair and objective review.
